# Differences in pulse oximetry saturation from sea level to high altitude among subjects with and without respiratory disease. A population-based, cross-sectional study

**DOI:** 10.3389/fphys.2026.1956566

**Published:** 2026-10-26

**Authors:** Mauricio Gonzalez-Garcia, Carlos A. Torres-Duque, Carlos Aguirre-Franco, Abraham Alí-Munive, Nadia Juliana Proaños, Carolina Perez, Alejandro Casas-Herrera

**Affiliations:** 1 Fundación Neumológica Colombiana, Bogotá, Colombia; 2 Universidad de La Sabana, Chia, Colombia; 3 Department of Radiology. Fundación Cardioinfantil, Bogotá, Colombia

**Keywords:** altitude (MeSH), cross-sectional studies, healthy (MeSH), hypoxia, oximetry, oxygen saturation (SO2), pulmonary disease, reference values (MeSH)

## Abstract

**Introduction:**

Oxygen saturation (SpO_2_) is used to detect hypoxemia and guide oxygen therapy, but its interpretation varies with altitude, age, and lung disease. Although >500 million people live at ≥1,500 m, population-based data comparing SpO_2_ across altitudes in adults with and without chronic respiratory disease are scarce. We aimed to compare SpO_2_ between these groups from sea level to 3,030 m, identify factors associated with SpO_2_, and describe SpO_2_ by altitude and age in adults without respiratory disease. We hypothesized that SpO_2_ would be lower in participants with respiratory disease, with differences increasing with altitude.

**Methods:**

PREPOCOL II is a population-based, cross-sectional study with a probabilistic, two-stage, clustered sampling design to estimate the prevalence and risk factors for respiratory disease among adults ≥40 years in Colombian cities at different altitudes (18-3030 m). All participants completed a standardized questionnaire, spirometry, and SpO_2_ measurement. Participants with respiratory disease (RDP), including chronic obstructive pulmonary disease, asthma, chronic bronchitis, obstructive sleep apnea, and interstitial lung disease, were compared with controls using Mann-Whitney and Kruskal-Wallis tests, multivariable linear regression, and comparisons of altitude coefficients between groups.

**Results:**

A total of 1146 RDP and 4613 controls were included. RDP were older, and had lower BMI and lung function. SpO_2_ was lower in RDP than in controls at all altitudes and decreased in both groups as altitude and age increased (p<0.001). In multivariable models, altitude showed the strongest association with SpO_2_ (standardized β −0.69 in controls and −0.71 in RDP), followed by age and BMI (and FEV_1_ in RDP). The decrease in SpO_2_ per unit of altitude was larger in RDP (B −1.15 vs. −0.80; difference p<0.001), and the between-group difference became evident at ~1,500 m (median 97.0% vs. 95.5%).

**Conclusion:**

Among lifelong residents living at altitudes from sea level to 3,030 m, SpO_2_ was lower in RDP than in controls. SpO_2_ decreased with altitude and age in both groups, with altitude being the variable most strongly associated with SpO_2_. SpO_2_ values should be interpreted in relation to specific altitude and age. In RDP, the lower SpO_2_ was most evident at altitudes of ~1,500 m and above.

## Introduction

Pulse oximetry saturation (SpO_2_) is a safe, simple, continuous, and noninvasive method for estimating arterial oxygen saturation (SaO_2_). SpO_2_ measurement is widely used across many settings, including critical care, hospital wards, outpatient settings, sleep laboratories, exercise laboratories, pulmonary rehabilitation, and other settings. SpO_2_ decreases in many clinical conditions, including respiratory and cardiovascular disease, sleep-related respiratory disturbances, and obesity. In healthy subjects, SpO_2_ decreases mainly with age and altitude (Pretto et al., 2014). At sea level, the normal SpO_2_ range in healthy adults is approximately 96–98%, and most clinical thresholds for detecting hypoxemia and prescribing oxygen have been derived from sea-level populations (O'driscoll et al., 2017).

Chronic respiratory diseases affected an estimated 545 million people worldwide in 2017, with chronic obstructive pulmonary disease (COPD) and asthma accounting for most of this burden (Collaborators, 2020). In Latin America, population-based studies using post-bronchodilator spirometry have reported COPD prevalence of 7.8–19.7% among adults ≥40 years (Menezes et al., 2005) and 8.9% in five Colombian cities at different altitudes in the first PREPOCOL study (Caballero et al., 2008). The pooled analysis of PREPOCOL, PLATINO, BOLD, and EPI-SCAN found no independent association between altitude and COPD prevalence but showed a higher proportion of undiagnosed disease among high-altitude residents (Horner et al., 2017).

Elevations below 1500 m are considered low altitude; elevations ≥ 1,500 m and < 2,500 m are considered moderate altitude; and elevations > 2,500 m (~8200 ft) are considered high altitude (Barry and Pollard, 2003). Based on georeferenced population and elevation data, a recent study estimated that more than 500 million people live at ≥1,500 m, 81.6 million at ≥2,500 m, and 14.4 million at ≥3,500 m, with a large share of these inhabitants in Latin American countries (Tremblay and Ainslie, 2021). As altitude increases, barometric pressure (BP) decreases, and consequently, inspired oxygen pressure (PIO_2_), arterial oxygen pressure (PaO_2_), and SpO_2_ also decrease. Although physiological responses to hypobaric hypoxia begin at lower altitudes, they are most pronounced above 2500 m, and the risk of developing altitude sickness is higher (Luks et al., 2019; Luks and Hackett, 2022).

Reference SpO_2_ values for residents at altitude have been reported in Andean and Himalayan populations. In Peru, SpO_2_ decreased progressively from sea level to 5,100 m, with a steeper decline above 2,500 m (Rojas-Camayo et al., 2018). Among Aymara residents at 3,900–4,000 m, mean SpO_2_ was ~92% (Beall et al., 1999). Arterial blood gas (ABG) reference values have also been reported for healthy adults at 1,400 m (Crapo et al., 1999), 2,240 m (Cid-Juarez et al., 2023), and 2,640 m (Gonzalez-Garcia et al., 2020). In COPD patients residing at altitude in Tibet and Xinjiang, mean SpO_2_ fell from 93.5% at 2,100–3,000 m to 86.3% above 4,000 m (Guo et al., 2020). In a systematic review of acute altitude exposure in COPD patients, PaO_2_ decreased by 6.3 mmHg per 1,000 m of altitude (Sevik et al., 2025).

However, these studies have important gaps. Population-based studies at different altitudes either did not distinguish between subjects with and without respiratory disease (Perez-Padilla et al., 2006) or were limited to COPD patients living at high altitude (Guo et al., 2020). The evidence on how chronic respiratory disease modifies the altitude-SpO_2_ relationship derives mainly from lowlanders acutely exposed to altitude (Furian et al., 2022), not from lifelong residents. To our knowledge, the SpO_2_ of lifelong residents with and without chronic respiratory disease has not been compared within the same population-based study, using identical methods, across a gradient from sea level to ~3,000 m. This information is clinically relevant: interpreting a given SpO_2_ value as normal or abnormal, deciding on further testing, and prescribing oxygen all depend on knowing the expected values for the patient’s altitude, age, and disease status. PREPOCOL II was an analytical, population-based, cross-sectional study in Colombia of 5,759 adults aged 40 years or older. Because all participants underwent the same standardized questionnaire, spirometry, and SpO_2_ measurement, this study offers an opportunity to address this gap.

The objectives of this study were to describe how altitude of residence, age, and chronic respiratory disease are associated with resting SpO_2_ in adults ≥40 years; to compare SpO_2_ between participants with and without chronic respiratory disease across altitudes from sea level to 3,030 m; to identify factors independently associated with SpO_2_ in each group; and to describe SpO_2_ by altitude and age in participants without respiratory disease. We explored whether SpO_2_ would be lower in participants with respiratory disease than in controls at all altitudes and whether the between-group difference would increase with altitude.

## Materials and methods

### Study design and participants

PREPOCOL II is an analytical, population-based, cross-sectional study whose primary objective was to determine the prevalence and risk factors for respiratory disease among adults aged 40 years or older. Trained field staff collected data at participants’ homes from 2022 to 2024.

The study frame was the non-institutionalized individuals aged ≥40 years living in nine participating municipalities. The sample size was calculated based on the primary objective of estimating the prevalence of COPD and asthma, using PREPOCOL I data (Caballero et al., 2008). With a 1.5 design-effect adjustment, an accepted 5% type I error rate, and a 3% sampling variability relative to the population parameter, the estimated minimum sample size was 5,600 subjects: 800 per urban city and 400 per rural municipality. Subjects were selected using a probabilistic, two-stage clustered sampling technique; sectors were randomized using official maps from the Colombian National Statistics Department.

Finally, the study included 5,759 adults aged 40 years or older, of both sexes, with lifelong residence in nine cities ranging from sea level to high altitude: 819 in Barranquilla (18 m), 448 in San Estanislao (26 m), 425 in Santa Rosa (43 m), 850 in Bucaramanga (959 m), 816 in Cali (1,018 m), 785 in Medellín (1,495 m), 807 in Bogotá (2,640 m), 398 in Samacá (2,765 m), and 411 in Aquitania (3,030 m).

For analysis, the three municipalities below 50 m were grouped. Between 18 and 43 m, the estimated BP differs by only ~2 mmHg (758 vs. 756 mmHg) and PIO_2_ by <0.5 mmHg (148.8 vs. 148.4 mmHg) (West, 1996), a difference without physiological relevance for SpO_2_. We excluded subjects in hospitals, prisons, or nursing homes; those with neurological, mental, or emotional conditions that precluded reliable information or acceptable spirometry maneuvers; and those with contraindications to salbutamol administration. The study was approved by the Institutional Research Ethics Committee (Approval Document No. 202103-26211), and participants provided informed consent.

To assess differences in SpO_2_, the total population was divided into two groups: participants with respiratory disease (RDP) and those without respiratory disease (control). The RDP group included subjects with chronic obstructive pulmonary disease (COPD), asthma, chronic bronchitis (CB), obstructive sleep apnea (OSA), and interstitial lung disease (ILD). The control group included subjects without these conditions.

### Measurements

Each subject completed an interviewer-administered respiratory health questionnaire adapted from PREPOCOL I (Caballero et al., 2008) and PLATINO (Menezes et al., 2005), covering demographic data, respiratory symptoms, risk factors for respiratory disease, and antecedents of respiratory disease.

Spirometry was performed by a trained technologist before and after 200 µg of inhaled salbutamol, using a portable ultrasonic spirometer (EasyOne; ndd Medical Technologies, Zurich, Switzerland) in accordance with the American Thoracic Society and European Respiratory Society recommendations (Graham et al., 2019). Per the manufacturer’s instructions, the device’s flow sensor does not require user calibration. All spirometry included daily quality control, and only maneuvers meeting ATS/ERS acceptability and repeatability criteria (grades A–C) were analyzed. Reference equations were the Global Lung Function Initiative 2012 multi-ethnic equations (Quanjer et al., 2012).

SpO_2_ was measured with a fingertip pulse oximeter (Onyx^®^ Vantage 9590; Nonin Medical Inc., Plymouth, MN, USA) before bronchodilator administration in all participants. Pulse oximeters are factory-calibrated against arterial SaO_2_ and cannot be user-calibrated; the manufacturer reports an accuracy of ±2 digits for SpO_2_ 70–100%. Measurements were taken after 5 minutes of seated rest, breathing room air, without nail polish, and only when a stable pulse signal was obtained. The average of six measurements taken at 10-s intervals was reported (Beall et al., 1999; Perez-Padilla et al., 2006).

### Definitions

We used epidemiological definitions for COPD, asthma, and CB. COPD was defined as a post-bronchodilator FEV_1_/FVC < 0.70 (Gold, 2026); asthma: two or more episodes of wheezing and dyspnea with normal post-β2 spirometry (Gonzalez-Garcia et al., 2015); CB: cough and expectoration for three or more months in two consecutive years (Council, 1965). OSA diagnosis was based on self-report, with an affirmative answer to the question: “Have you been diagnosed with sleep apnea using a polysomnogram?” Subjects with ILD were identified using a stepwise strategy: those with a possible restrictive pattern on spirometry or self-reported ILD were invited to undergo additional studies, including diffusion capacity, lung volumes, and high-resolution CT of the lungs. The ILD diagnosis was confirmed by the ILD Multidisciplinary Discussion (MDD), which included two pulmonologists (CT, MG) and a radiologist (CP) with expertise in ILD, following the international guidelines (Ryerson et al., 2025; Raghu et al., 2022).

### Statistical analysis

Normality of variables was assessed using the Kolmogorov-Smirnov test. Continuous variables were reported as mean ± standard deviation (SD) if normally distributed, or as median and interquartile range otherwise. Qualitative variables were reported as proportions. Characteristics of RDP and controls were compared using the t-test, Mann-Whitney test, and the Chi-square test. To assess differences in SpO_2_ between the RDP and control groups, we used the Mann-Whitney test. For differences by altitude and age, we used the Kruskal-Wallis test. To establish SpO_2_ by altitude and age in the control group, the 5th and 95th percentiles were used as the lower (LL) and upper (UL) limits of normality.

Factors associated with SpO_2_ were assessed using separate multivariable linear regression models in controls and RDP. Candidate variables, selected *a priori* on physiological grounds, were age, sex, BMI, altitude, smoking, wood-smoke exposure, and FEV_1%_ predicted; variables were selected using a stepwise procedure. To assess whether respiratory disease modified the association between altitude and SpO_2_, the regression coefficients for altitude from the separate models for controls and RDP were compared using a z-test for the difference between two independent estimates (Knol and Vanderweele, 2012; Altman and Bland, 2003); the same approach was applied to age and BMI. Altitude was coded identically in both models. Model assumptions were verified with residual plots (linearity and homoscedasticity) and variance inflation factors.

Two-tailed hypotheses were tested at the 0.05 level of significance, and 95% CIs are reported for regression coefficients. Statistical analyses were conducted using IBM SPSS Statistics for Windows, version 25.0 (IBM Corp., Armonk, NY, USA).

## Results

### Subjects characteristics

A total of 5759 subjects, including 1146 with respiratory disease (RDP group) and 4613 without respiratory disease (control group), aged 40 to 103 years (mean 59.9 ± 11.6 years), with 60.7% female, were included. RDP were older, had greater exposure to tobacco and wood smoke, and had lower body mass index (BMI) and lung function. Among the RDP, 697 had COPD (60.8%), 293 had asthma (25.6%), 88 had CB (7.7%), 54 had OSA (4.7%), and 14 had ILD (1.2%). The distribution of RDP and controls by altitude is shown in **Table 1**. The proportion of RDP (19.9%) reflects the prevalence of these diseases in the sampled population.

**Table 1:** Subject characteristics and arterial oxygen saturation by group diagnosis (N = 5759).

	Controls N=4613	RDP N=1146	p
Age, years	58.7 ± 11.2	64.8 ± 12.2	<0.001
Female sex	2917 (63.2)	579 (50.5)	<0.001
Smoking	1202 (26.1)	504 (44.0)	<0.001
Exposure to wood smoke	1514 (32.8)	448 (39.1)	<0.001
BMI, Kg/m^2^	27.4 ± 4.7	26.8 ± 5.1	0.001
FVC, L	3.17 ± 0.84	2.94 ± 0.90	<0.001
FVC, % of predicted	96.3 ± 15.2	92.1 ± 19.8	<0.001
FEV_1_, L	2.50 ± 0.67	1.97 ± 0.73	<0.001
FEV_1_, % of predicted	95.8 ± 15.5	78.7 ± 22.4	<0.001
FEV_1_/FVC	0.79 ± 0.05	0.66 ± 0.12	<0.001
SpO_2_, % by altitude (m)			
• < 50 m	98.0 (97.7 - 98.5)	98.0 (97.0 - 98.0)	<0.001
• 959 m	97.7 (96.5 - 98.3)	97.0 (95.0 - 98.0)	<0.001
• 1018 m	97.3 (96.0 - 98.0)	97.0 (95.7 - 97.7)	<0.001
• 1495 m	97.0 (95.8 - 97.8)	95.5 (93.0 - 97.1)	<0.001
• 2640 m	93.2 (91.5 - 95.0)	91.6 (89.8 - 93.3)	<0.001
• 2765 m	91.8 (90.5 - 93.8)	90.7 (88.5 - 92.5)	<0.001
• 3030 m	91.5 (89.7 - 93.7)	90.2 (87.5 - 92.2)	<0.001

RDP, Participants with respiratory disease; BMI, body mass index; FVC, forced vital capacity; FEV_1_, forced expiratory volume in the first second; SpO_2_, oxygen saturation.

Values reported as mean ± SD, median P_25–75_ or N (%)

### SpO_2_ differences by altitude

SpO_2_ was lower in RDP than in control subjects at all altitudes and decreased with increasing altitude (p<0.001) (**Table 1**; **Figure 1**). In RDP, SpO_2_ began to decrease at lower altitudes than in controls, particularly among those >50 years, with the lowest values at ~3,000 m in subjects ≥70 years. The between-group difference in median SpO_2_ was ≤0.7 percentage points below 1,100 m and 1.1–1.6 points from 1,495 m upward (**Table 1**).

**Figure 1:**
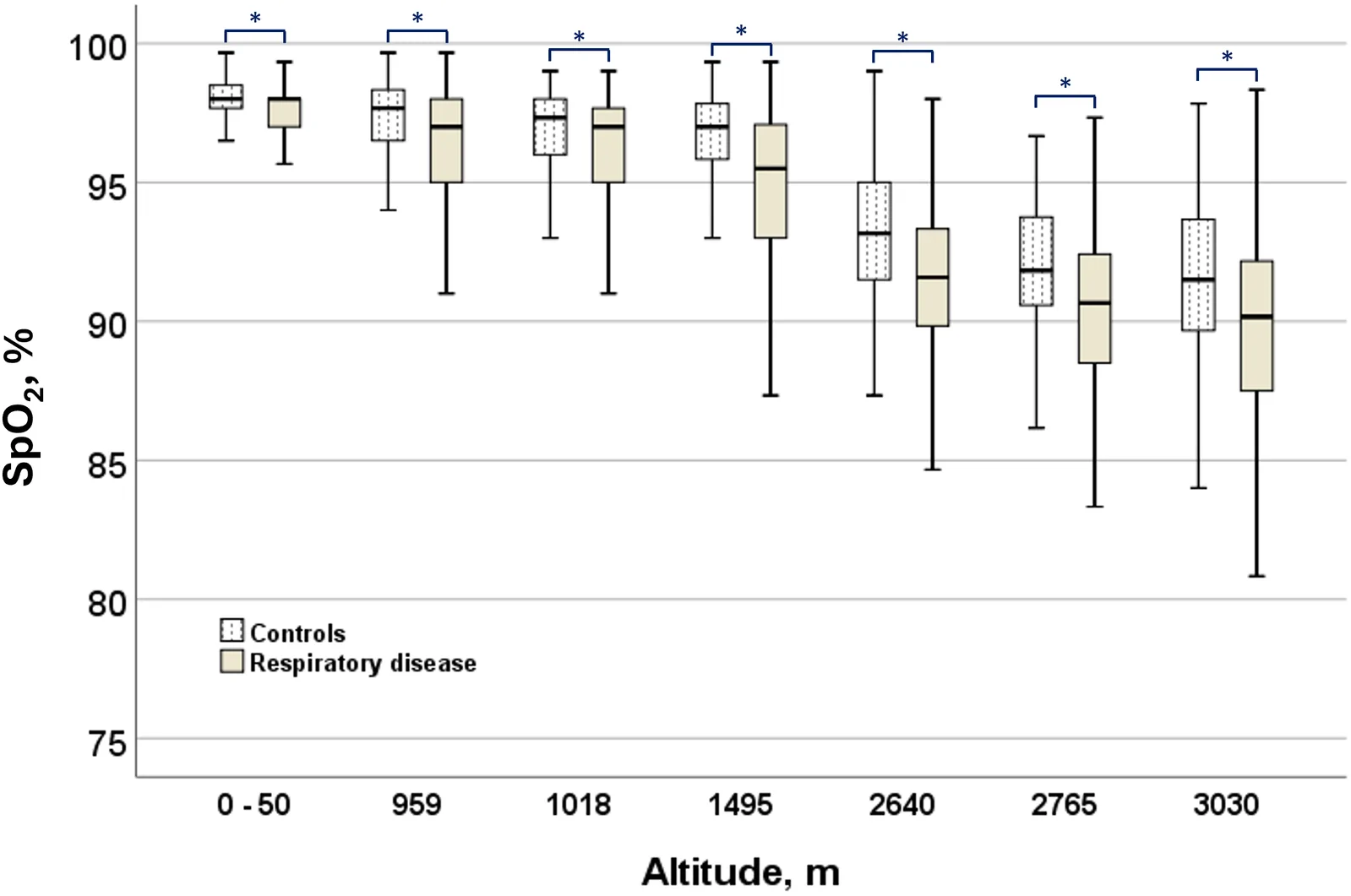
Differences in pulse oximetry saturation (SpO_2_) at different altitudes. SpO_2_ was lower in RDP than in control subjects at all altitudes, and in both groups, SpO_2_ decreased with increasing altitude (p<0.001). *p<0.05 differences between controls and subjects with respiratory disease; p<0.001 differences by altitude in controls and subjects with respiratory disease.

In control subjects, SpO_2_ also decreased significantly with age (p<0.001), with lower values among those living in high-altitude cities (**Table 2**; **Figure 2A**). **Table 2** presents the median and limits of normality for SpO_2_ by age and city altitude in subjects without respiratory disease. Because COPD and asthma were the most frequent respiratory diseases in the RDP group, with participants across all altitudes and ages, we show in **Figure 2B** the changes in SpO_2_ by age and altitude in this RDP subgroup (n = 990).

**Table 2:** SpO_2_ by age and altitude in subjects without respiratory disease (control Group) (N = 4613).

City (altitude, m)	Subjects (N)	Age, years				p
		40-49	50-59	60-69	≥ 70	
< 50	1442	98.0 (96.0 - 99.0)	98.0 (96.2 - 99.0)	98.0 (96.0 - 99.0)	98.0 (96.0 - 99.0)	0.001
959	713	98.0 (95.5 - 99.0)	97.5 (95.0 - 99.0)	97.3 (94.0 - 99.0)	97.2 (93.0 - 99.0)	<0.001
1018	687	97.8 (95.5 - 99.0)	97.5 (95.0 - 98.3)	97.0 (94.0 - 98.7)	96.9 (93.7 - 98.0)	<0.001
1495	637	97.3 (95.0 - 99.0)	97.0 (94.0 - 98.5)	96.7 (92.7 - 98.4)	96.8 (92.3 - 98.2)	<0.001
2640	621	94.2 (90.8 - 97.8)	93.5 (89.7 - 96.8)	93.0 (89.7 - 96.7)	92.0 (87.8 - 96.2)	<0.001
2765	243	93.0 (90.3 - 95.7)	91.8 (89.3 - 95.3)	91.2 (86.2 - 95.2)	89.7 (87.3 - 94.2)	<0.001
3030	270	93.1 (89.5 - 97.3)	91.7 (87.3 - 95.7)	90.2 (85.8 - 94.5)	90.3 (84.0 - 95.0)	<0.001
P*		<0.001	<0.001	<0.001	<0.001	

SpO_2_, pulse oximetry saturation. Values reported as median and P_5-95._

P = differences in SpO_2_ by age group at each altitude. P* = differences in SpO_2_ by altitude within each age group

**Figure 2:**
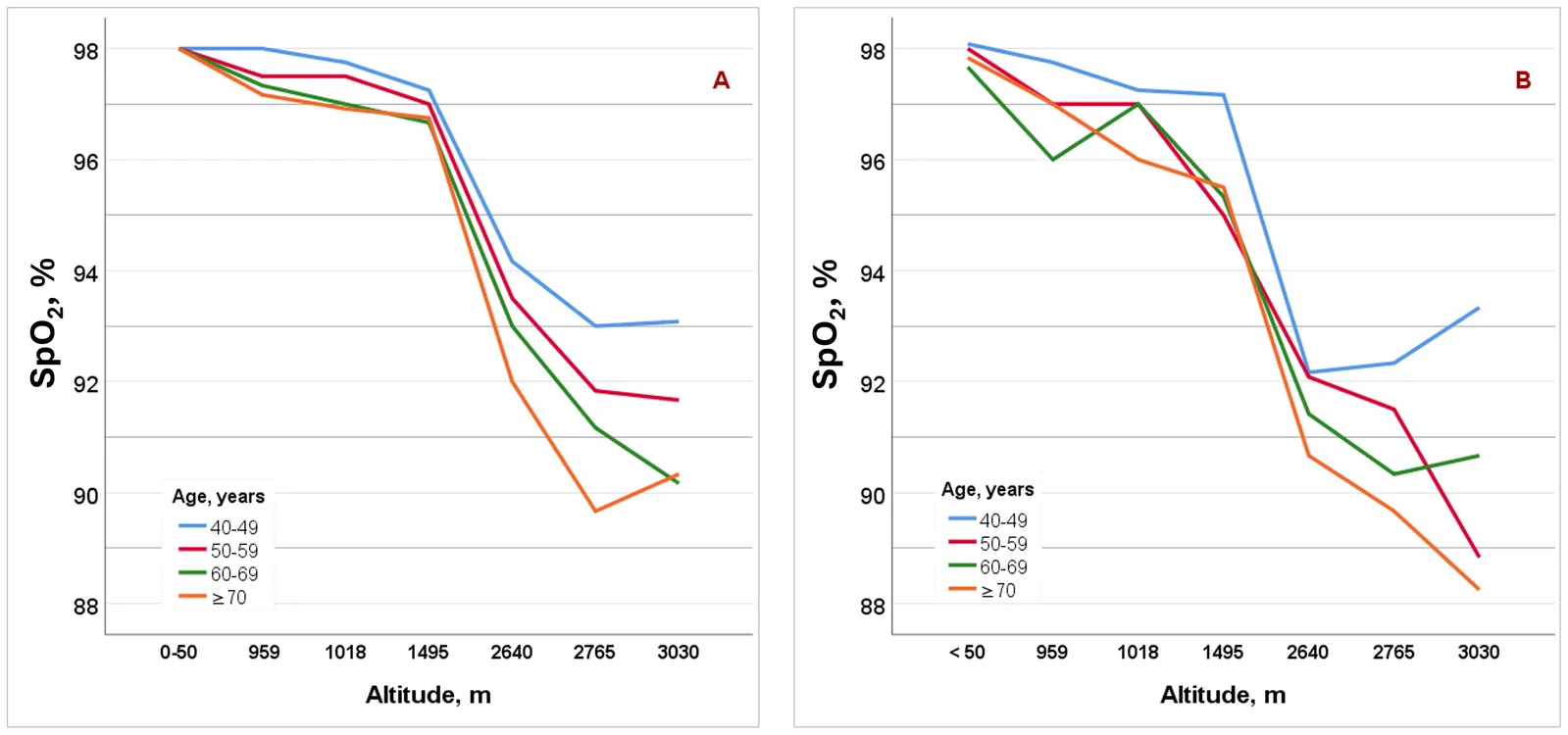
Pulse oximetry saturation (SpO_2_) by age and altitude in subjects without respiratory disease **(A)** and in subjects with asthma and COPD **(B)**. In control subjects **(A)**, SpO_2_ decreased significantly with increasing altitude and age (p<0.001), with lower values in older individuals living in high-altitude cities. In participants with asthma and COPD **(B)**, SpO_2_ also decreased significantly with increasing altitude and age (p<0.001) but began to decline at lower altitudes than in controls.

### Multivariable analysis

In the multivariable linear regression analysis (**Table 3**), the variables independently associated with SpO_2_ in control subjects were age, BMI, and altitude (R² = 0.495). Each unit of increased altitude was associated with a 0.80-point lower SpO_2_ (95% CI −0.83 to −0.78); each additional decade of age with a 0.32-point lower SpO_2_ (95% CI −0.37 to −0.27); and each 5 kg/m² of BMI with a 0.25-point lower SpO_2_ (95% CI −0.31 to −0.19). Altitude had the largest standardized coefficient (β = −0.69). In RDP, the variables associated with SpO_2_ were age, BMI, altitude, and FEV_1_ % predicted (R² = 0.534): −1.15 points per unit of altitude (95% CI −1.22 to −1.09), −0.50 per decade of age (95% CI −0.64 to −0.37), −0.54 per 5 kg/m² of BMI (95% CI −0.70 to −0.38), and +0.28 per 10% predicted of FEV_1_ (95% CI 0.21 to 0.36); altitude again had the largest standardized coefficient (β = −0.71). Sex, smoking, and wood-smoke exposure were not retained in either model.

**Table 3:** Multivariate analysis for SpO_2_ in control subjects and respiratory disease participants.

Groups		Unstandardized coefficients		Standardized coefficients	p	95,0% confidence interval for B	
		B	Std. error	Beta		Lower bound	Upper bound
Control group (p<0.001, R^2^ = 0.495)	(Constant)	102.297	0.243		<0.001	101.820	102.773
	Age, years	-0.032	0.003	-0.128	<0.001	-0.037	-0.027
	BMI, Kg/m^2^	-0.050	0.006	-0.085	<0.001	-0.062	-0.038
	Altitude	-0.803	0.012	-0.689	<0.001	-0.827	-0.779
RDP group (p<0.001, R^2^ = 0.534)	(Constant)	103.027	0.761		<0.001	101.533	104.520
	Age, years	-0.050	0.007	-0.151	<0.001	-0.064	-0.037
	BMI, Kg/m^2^	-0.107	0.016	-0.135	<0.001	-0.139	-0.075
	Altitude	-1.152	0.033	-0.708	<0.001	-1.217	-1.086
	FEV_1_, % predicted	0.028	0.004	0.157	<0.001	0.021	0.036

RDP, Participants with respiratory disease; BMI, body mass index; FEV_1_, forced expiratory volume in the first second.

The association between altitude and SpO_2_ was steeper in RDP than in controls (difference in B −0.35, 95% CI −0.42 to −0.28; p<0.001), indicating effect modification by respiratory disease. The associations with age (difference −0.018 per year, 95% CI −0.033 to −0.003; p=0.018) and BMI (difference −0.057 per kg/m², 95% CI −0.090 to −0.024; p<0.001) were also stronger in RDP.

## Discussion

The main findings of this population-based study of lifelong residents living from sea level to 3,030 m were: (1) SpO_2_ was lower in participants with chronic respiratory disease than in controls at all altitudes; (2) in both groups SpO_2_ decreased with increasing altitude and age, and altitude was the variable most strongly associated with SpO_2_ after adjustment for age and BMI (and FEV_1_ in RDP); (3) the difference between groups became evident from ~1,500 m, and the association between altitude and SpO_2_ was steeper in RDP; and (4) we describe SpO_2_ values by altitude and age in adults ≥40 years without respiratory disease.

Compared with sea level, studies using ABG have reported lower PaO_2_ and SaO_2_ among healthy inhabitants at low (Crapo et al., 1999), moderate (Cid-Juarez et al., 2023), and high altitude (Gonzalez-Garcia et al., 2020). In Peru, among subjects aged 1 to 80 years, SpO_2_ decreased across a wide range of altitudes, with a more pronounced decline above 2,500 m (Rojas-Camayo et al., 2018). In healthy, unacclimatized lowlanders acutely exposed to altitude, a meta-analysis found a decline in PaO_2_ of ~12 mmHg per 1,000 m (Forrer et al., 2023). Our control values at 2,640–3,030 m (SpO_2_ medians 91.5–93.2%) are consistent with these reports. Because no city between 1,495 and 2,640 m was included, our data cannot pinpoint the altitude at which SpO_2_ begins to decline sharply; however, they show that between-group differences were already evident at 1,495 m.

The steeper altitude–SpO_2_ slope in RDP and the divergence from ~1,500 m are consistent with the sigmoid shape of the oxyhemoglobin dissociation curve. Subjects with airway disease have ventilation-perfusion mismatch and therefore a lower PaO_2_ at the same PIO_2_. As PIO_2_ decreases with altitude, their PaO_2_ reaches the steep portion of the curve at a lower altitude than in healthy subjects, so the same decrease in PaO_2_ produces a larger fall in SpO_2_ (Furian et al., 2022). This aligns with the meta-analysis of patients with COPD exposed to altitude, in which the absolute decrease in PaO_2_ per 1,000 m was smaller than in healthy subjects but starting from a lower baseline PaO_2_ (Sevik et al., 2025), and with our previous observation of greater gas-exchange impairment in COPD patients residing at 2,640 m (Gonzalez-Garcia et al., 2021). This interpretation should be confirmed with ABG, which were not measured in this study.

As altitude increases, BP decreases, and consequently, PIO_2_, PaO_2_, and SpO_2_ also decrease. Increased ventilation with decreased arterial carbon dioxide pressure (PaCO_2_) is a well-recognized compensatory mechanism for adaptation to altitude (West, 2004; Dempsey and Forster, 1982; Pamenter and Powell, 2016). Studies of residents in the Andes and Tibet document population differences in adaptation by race (Beall, 2007; Moore, 2017), with higher resting ventilation and increased ventilatory response to hypoxia in Tibetans, and higher hemoglobin concentrations in the Andeans at the same altitude (Gassmann et al., 2019). ABG studies in Mexico at moderate altitude (Cid-Juarez et al., 2023) and a previous study by our group in Bogota, Colombia, at high altitude (Gonzalez-Garcia et al., 2020) have shown lower PaO_2_ and SaO_2_ than at sea level, along with significantly lower PaCO_2_ values, indicating that increased alveolar ventilation is the main compensatory mechanism of adaptation to altitude in these subjects. Since the participants of the present study were lifelong residents, their SpO_2_ values reflect chronic (not acute) adaptation, which explains why SpO_2_ at 2,640–3,030 m was higher than that reported in lowlanders acutely exposed to similar altitudes (Dunnwald et al., 2021).

As with altitude, previous ABG studies have also shown decreases in PaO_2_ and SaO_2_ with age (Cerveri et al., 1995; Crapo et al., 1999; Klaestrup et al., 2011; Gonzalez-Garcia et al., 2020), parallel to an increase in P(A-a)O_2_ and may be related to increased pulmonary ventilation-perfusion imbalance (Cardus et al., 1997) and diffusion limitation in elderly subjects (Guenard and Marthan, 1996). In our controls, the effect of age was small at sea level, where medians were 98% in all age groups, but became progressively larger with altitude (**Table 2**), which is again consistent with the position on the oxyhemoglobin dissociation curve.

Besides altitude and age, BMI was also associated with lower SpO_2_ in both RDP and controls. As in our findings, other studies have reported lower SpO_2_ in obesity (Cid-Juarez et al., 2023; Kapur et al., 2013; Littleton and Tulaimat, 2017), which is likely associated with reduced pulmonary compliance, restrictive ventilatory impairment, peripheral airways disease and air trapping, and increased ventilation/perfusion mismatch (Dixon and Peters, 2018).

Consistent with our results, another population-based cross-sectional study in cities at low and moderate altitudes, without distinguishing between healthy subjects and those with respiratory disease, found that the factors associated with SpO_2_ were altitude, age, BMI, and FEV_1%_ of predicted (Perez-Padilla et al., 2006). In our population, FEV_1_ was not related to SpO_2_ in controls, in whom FEV_1_ was mostly within the normal range, but it was in the RDP group, as expected, given that most participants in this group had obstructive airway disease; each 10% decrease in FEV_1%_ predicted was associated with a ~0.3-point lower SpO_2_. This aligns with the effects of established airflow limitation on gas exchange (Gold, 2026), with greater impact in COPD patients living at high altitude (Gonzalez-Garcia et al., 2021), and with a six-year follow-up cohort of adults in which the decline in SpO_2_ was associated with low FEV_1_, a history of smoking, and a higher BMI (Vold et al., 2014).

Although sex differences in SpO_2_ have been reported, we did not observe them in this population of adults over 40 years of age. This may be because the reported sex differences have been observed in younger populations (Levental et al., 2018; Klaestrup et al., 2011). In a previous ABG study by our group in healthy subjects residing at high altitude, young women had higher PaO_2_ and SaO_2_ and lower PaCO_2_, which we attributed to hormonal effects, especially progesterone, a known respiratory stimulant (Gonzalez-Garcia et al., 2020). Most women in the present study were peri- or postmenopausal (mean age of controls 58.7 years), which may explain the absence of sex differences. Moreover, recent studies at high altitude found no effect of menstrual cycle phase on resting SpO_2_ (Tagliapietra et al., 2024; Citherlet et al., 2025). PREPOCOL II was designed to estimate the prevalence of COPD and other chronic respiratory diseases, which are uncommon before age 40. Restricting the sample to adults ≥40 years is standard in international COPD prevalence studies (Caballero et al., 2008; Menezes et al., 2004) and maximizes the number of cases. Consequently, the SpO_2_ values reported here apply to adults ≥40 years and should not be extrapolated to younger people, for whom SpO_2_ is expected to be higher.

Clinical implications. Our results suggest that: (1) a “normal” SpO_2_ depends on where the patient lives: in healthy adults ≥40 years, the median SpO_2_ was 98% at sea level but 91.5–93% at 2,640–3,030 m, and the 5th percentile fell to 84–90% in those ≥60 years (**Table 3**); therefore, sea-level cut-offs should not be applied unchanged at altitude, and values below the altitude- and age-specific 5th percentile warrant additional clinical evaluation; (2) patients with chronic respiratory disease living at ≥1,500 m have lower SpO_2_ than their peers without disease, and the difference increases with age, supporting routine SpO_2_ measurement in these patients, including those living at intermediate altitudes; and (3) for guideline developers and health planners, criteria for oxygen prescription and referral derived at sea level should be validated in high-altitude populations, where a larger proportion of patients may meet these criteria.

To our knowledge, few population-based studies have compared SpO_2_ among subjects with and without chronic respiratory disease at different altitudes within the same study. Strengths of this study include a large, probabilistically selected sample; inclusion of subjects across a wide age range (40 to 103 years) at altitudes from sea level to 3,030 m; classification of respiratory diseases using definitions accepted in epidemiological studies; and inclusion of variables related to SpO_2_ that have been used in previous studies. Trained personnel performed spirometry using spirometers that met the required technical standards, with daily quality control (Graham et al., 2019), and SpO_2_ was measured with a validated pulse oximeter using a standardized protocol.

Our study has several limitations. First, the cross-sectional design allows identification of associations but not causal inference; however, lifelong residence altitude precedes SpO_2_ measurement, making reverse causation implausible for this variable. Second, moderate altitude (1,500–2,500 m) was not represented: Medellín (1,495 m) lies at the lower limit of this category, and no city between 1,495 and 2,640 m was included, so the shape of the SpO_2_ curve in this range could not be determined. Third, as in other population-based studies, misclassification is possible. Although we used a questionnaire derived from standardized instruments, performed standardized pulmonary function measurements, employed accepted definitions of COPD, asthma, and CB, and identified subjects with ILD through multidisciplinary discussion, other lung diseases may have been missed because of the limitations of the tools used or participants’ lack of awareness of their condition. OSA, identified by self-report of a polysomnographic diagnosis, is largely underdiagnosed (Benjafield et al., 2019; Young et al., 1997); undiagnosed cases in the control group would bias the between-group differences toward the null effect. Fourth, using a validated pulse oximeter, known limitations of pulse oximetry, including overestimation in darker skin pigmentation, cold-induced vasoconstriction, and other situations (Sjoding et al., 2020; Pretto et al., 2014; Dunnwald et al., 2021), could have affected the results. The standardized protocol and the averaging of six readings reduce, but do not eliminate, these sources of error. Fifth, ABG and hemoglobin were not measured, so the proposed physiological mechanisms remain inferences. Sixth, comorbidities such as heart failure or anemia were not recorded.

Future studies should include populations living between 1,500 and 2,500 m to define the altitude at which SpO_2_ begins to decline steeply, particularly in patients with chronic respiratory disease; explore differences in SpO_2_ across respiratory diseases; derive continuous, altitude-, age-, and sex-specific reference equations for SpO_2_ in adults; follow PREPOCOL II participants longitudinally to determine whether altitude-adjusted SpO_2_ predicts exacerbations, need for oxygen therapy, and mortality; and validate SpO_2_ and PaO_2_ thresholds for long-term oxygen prescription at altitude using ABG; and assess nocturnal and exercise oxygen saturation in residents with COPD at different altitudes.

In conclusion, in this population-based study of lifelong residents living from sea level to 3,030 m, SpO_2_ was lower in adults with chronic respiratory disease than in those without at all altitudes, and in both groups, SpO_2_ decreased with increasing altitude and age. After adjusting for age, BMI, and FEV_1_, altitude was the variable most strongly associated with SpO_2_, and the difference between subjects with and without respiratory disease became evident from ~1,500 m. The altitude and age-specific SpO_2_ values reported for adults without respiratory disease can help clinicians interpret pulse oximetry in populations living at different altitudes.

## Data Availability

The raw data supporting the conclusions of this article will be made available by the authors, without undue reservation.
